# The Evolution of the Cephalometric Superimposition Techniques from the Beginning to the Digital Era: A Brief Descriptive Review

**DOI:** 10.1155/2021/6677133

**Published:** 2021-04-23

**Authors:** A. Lo Giudice, V. Ronsivalle, G. Zappalà, R. Leonardi, P. Campagna, G. Isola, G. Palazzo

**Affiliations:** Department of General Surgery and Surgical-Medical Specialties, School of Dentistry, University of Catania, Via S. Sofia 78, Catania 95124, Italy

## Abstract

Superimposition of craniofacial structures from radiographic examination has been always used for assessing changes in the maxilla-mandibular complexes, especially for the evaluation of potential changes occurring during growth as well as after orthodontic treatment and/or maxillofacial surgery. However, the availability of cone beam computed tomography (CBCT) and the recent advancement in 3D imaging have allowed the development of specific techniques for the registration and superimposition of virtual three-dimensional anatomical structures, improving the diagnosis and treatment plan strategies. In the present paper, it will be discussed the evolution of superimposition techniques from the beginning (2D) to the newest 3D approach, describing the most used methods and their main advantages and disadvantages, focusing primarily on accuracy and reproducibility of each technique.

## 1. Introduction

2Cephalometric superimposition is an analysis of lateral, frontal, or three-dimensional (3D) cephalograms of the same patient taken at different times. Since 1955, when Bjork [1] introduced this technique, cephalometric superimpositions gained growing importance due to the possibility to evaluate the patient's growth pattern between different ages, to evaluate changes in dentoalveolar and basal relationships after a course of orthodontic or surgical treatment, and to differentiate the changes due to growth and treatment [2].

In fact, superimpositions provide an overall assessment of the growth and treatment changes of the facial structures evaluating the amount and direction of maxillary and mandibular growth or displacement, changes in maxillary-mandibular relationship, and relative changes in soft tissue integument (nose, lips, and chin) and provide information on the overall displacement of the teeth [3].

There are different methods to superimpose two or more X-ray-derived datasets. Such methods can be classified in two main diagnostic approaches that have followed the enhancement of diagnostic imaging, i.e., two-dimensional (2D) and three-dimensional (3D) image techniques.

The early traditional method is a bidimensional (2D) evaluation based on the comparison of linear and angular measurements on serial superimpositions from cephalograms that have been taken at different times to evaluate the effect of growth or treatment. In this respect, tracings of the head films must be superimposed on relatively stable landmarks least affected by the growth, in order to be accurate and reproducible. Thus, superimpositions must be taken under identical conditions of magnification, head position, and radiological exposure, and tracing of the superimpositions must accurately locate the outlines or the relevant structures, eliminating confusing, unusable details.

Since lateral cephalogram is routinely used for the assessment of maxillofacial skeletal pattern as well as for the assessment of pre-post treatment changes, 2D cephalometric is still useful for orthodontists. However, conventional superimposition involves the representation of 3D complex structures, like the craniofacial complex, on a 2D flat X-ray film, with intrinsic limitation. Furthermore, 2D analysis has different drawbacks such as image distortion and magnification and difficulty in landmark identification due to the overlapping of the anatomical structures [4–7].

In order to overcome these disadvantages, nowadays, clinicians can refer to 3D imaging technology. In this respect, cone beam computed tomography (CBCT) represents the three-dimensional (3D) imaging method of choice in oral and maxillofacial fields [8]. Although multislice computed tomography (MSCT) presents higher resolution and higher contrast-to-noise ratio [9, 10], CBCT scans offer adequate images for diagnostic purpose, at lower risk of patients' radiation exposure [11–14]. Also, CBCT scans allow the rendering of anatomical dataset into a 3D anatomical model that could be used for linear, volumetric, and surface measurements, allowing a detailed analysis of the maxillofacial structures without magnification and distortion errors [12]. In particular, 3D rendering of maxilla and mandibular jaws is extremely useful for preoperative surgical planning and postoperative follow-up evaluation in orthognathic surgery and for the analysis of morphological, dimensional, and positional changes before and after orthodontic therapies [13, 14].

In the last years, a growing number of 3D superimposition techniques have been proposed by different authors using landmark-, surface-, or voxel-based registration. Each of these techniques allows the superimposition of consecutive CBCT-derived 3D models of the same patient in order to study the displacement and growth of the craniofacial complex, the facial asymmetries [15, 16], and the changes after an orthodontic or surgical treatment [17–22]. In the present paper, it will be discussed the evolution of superimposition techniques from the beginning (2D) to the newest 3D approach, describing the most used methods and their main advantages and disadvantages, focusing primarily on accuracy and reproducibility of each technique.

## 2. Materials and Methods

In this section, we provided and discussed a detailed description of the common methodologies used to perform superimposition of the craniofacial structures, according to the best available scientific contributions in orthodontic literature.

### 2.1. 2D Analysis

Cranial base structures have been used for superimpositions since both the neurocranium and its related cranial base achieve most of their growth potential at a relatively early age. Therefore, this part of the cranium is considered to be relatively stable. Several cranial axes were proposed in the past such as basion horizontal, broadbent triangle, basion-nasion plane, and sella-nasion line [23].

The areas that have to be evaluated include the changes in overall face, changes in the maxilla and its dentition, changes in the mandible and its dentition, amount and direction of condylar growth, and mandibular rotation.

**Figure 1:**
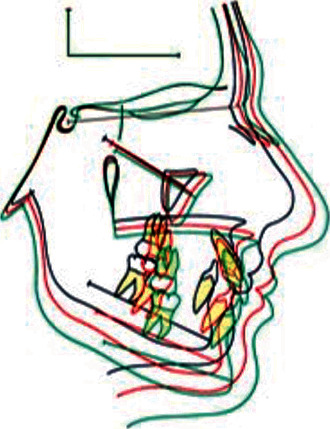
Example of 2D superimposition of cranial base structures.

Based on the American Board of Orthodontics color codes are used to facilitate identification of consecutive cephalograms (Figure 1):

- Pretreatment—black
- Progress—blue
- End of treatment—red
- Retention—green

Although these methods are easy to use, they have some disadvantages since they incorporate areas of the cranial base that may still change during growth stage such as the spheno-occipital synchondrosis or area of bone remodeling such as those recognizable by cephalometric points Nasion and Sella. Moreover, the position of the basion is influenced by the remodeling process on the surface of the clivus and on the anterior border of the foramen magnum and by the displacement of occipital bone due to growth at spheno-occipital synchondrosis [24].

Based on these considerations, Planché [25] created the best fit method of anterior cranial base identifying various bony surfaces in the anterior cranial base that are suitable for accurate superimpositions. They include the anterior wall of sella turcica, the contour of cribriform plate of ethmoid bone, details in trabecular system in ethmoid cells, median border of orbital roof, and plane of sphenoid bone.

Moreover, superimposition of maxillary and mandibular jaws was used to allow the evaluation of changes of specific bone structures such as the maxilla and the mandible.

The purpose of maxillary superimposition is to evaluate the movement of maxillary teeth in relation to the basal parts of maxilla. In this respect, some methods used the palatal plane [25] such as

- Superimposition along the palatal plane registered at ANS [26–30]
- Superimposition on the nasal floor with films registered at anterior surface of maxilla [31, 32]
- Superimposition along the palatal plane registered at pterygomaxillary fissure [27]

These methods are compromised because of the remodeling of the palatal shelves and the fact that the hard palate undergoes continuous resorption on its nasal surface and apposition on the oral side, making most of these methods of superimposition unsatisfactory. Furthermore, both ANS and PNS undergo significant anteroposterior remodeling.

Other methods superimpose on other structures such as

- The outline of infratemporal fossa and posterior portion of hard palate [33]
- Superimposition registering the maxilla on the common Pterygomaxillary, maintaining the basion horizontal relationship [34]
- Superimposition on the best fit of internal palatal structures [30]
- Superimposition on metallic implants on the anterior surface of the zygomatic process of the maxilla [35]

Similarly to the maxilla, mandibular areas can be superimposed in order to evaluate the movement of mandibular teeth in relation to the basal parts of the mandible.

There are different methods that use various landmarks such as

**Figure 2:**
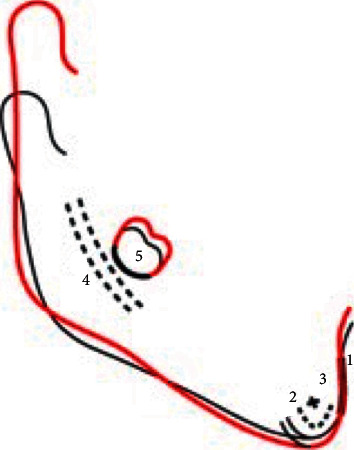
Example of 2D mandibular superimposition.

- Superimposition on the lower border of the mandible and on the inner table of symphysis [28]
- Superimposition on the mandibular plane
- Superimposition on anterior contour of the chin, the inner contour of the cortical plates at the inferior border of the symphysis and trabecular structure in the lower part of symphysis, the contour of mandibular canal, and the lower contour of mineralized molar germ (Figure 2)

### 2.2. 3D Analysis

The introduction of CBCT technology and the possibility to render a volumetric patient's dataset in a 3D virtual model have increased a growing interest by the scientific community in developing different methods to study and analyze the morphology and changes of the craniofacial complex using 3D superimposition methods. The main two technologies used to superimpose 3D images are surface-based registration and voxel-based registration. These techniques have introduced the possibility to superimpose two or more consecutive CBCT-derived 3D models of the same patient in order to study the growth of the craniofacial complex, to evaluate surgical or orthodontic treatment outcomes, to analyze the facial asymmetries, or to plan a surgical treatment [15, 16]. 3D registration techniques have different advantages with respect to 2D superimpositions. First of all, they well overcome the magnification, distortion, and landmark identification errors that frequently occur in 2D cephalometry [7]. Furthermore, the possibility to render a volumetric dataset in a 3D model of different anatomical structures (i.e., mandible, maxillary bone, and cranial base) allows to finely analyze the morphological changes of the whole craniofacial complex or of a specific anatomical area, giving the clinician a wider range of information with respect to traditional cephalometry. In addition to 2D superimpositions techniques, 3D registration methods permit to study the displacement of anatomical point in each direction of the space once a correct orientation of the head is preliminary achieved. Furthermore, using a 3D superimposition approach, it is possible to calculate the Euclidean distances between the surface of the registered 3D models and to highlight this deviation values in a colorimetric map using an iterative closest point evaluation method [36, 37]. As reported in literature, a good superimposition method should be able to register precisely and aid in understanding the changes as a result of growth and/or treatment relative to the structure of reference. According to previous studies, 3D superimposition techniques provide not only better accuracy and reliability with respect to 2D traditional techniques but also a better understanding of the morphological changes of the superimposed structures.

**Figure 3:**
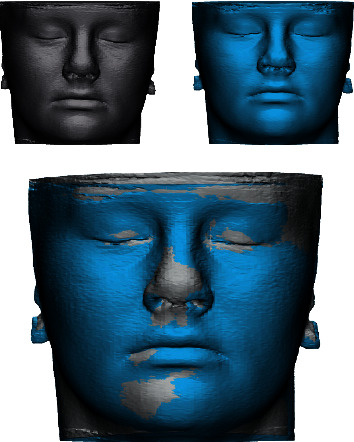
Example of surface-based superimposition of CBCT-derived patient's facial soft tissues before and after orthodontic treatment with maxillary expander.

The first method described in the literature for superimposition of 3D datasets is the surface-based registration (SBR). It was proposed by Hajeer et al. [36] in order to study the facial asymmetry before and after orthognathic surgery. This technique uses the surface of two or more segmented structures to superimpose them and requires a high-quality surface of 3D models for an accurate superimposition. It is also called “best fit” algorithm due to the iterative process (Iterative Closest Point (ICP) algorithm) which minimizes the surface distance between the two surfaces previously manually approximated (Figure 3). SBR calculated an estimation of the optimal rotational and translational movements between the surface of 3D models by minimizing the mean square distance between the models' mesh points. This distance is measured between a specified percentage of the points randomly selected on one 3D mesh and the corresponding 3D surface mesh. This technique, firstly proposed to study facial asymmetry, has been used by different authors in order to study the asymmetry and morphology of different anatomical districts such as the palatal vault, the mandible, the glenoid fossa, and other facial structures [37].

**Figure 4:**
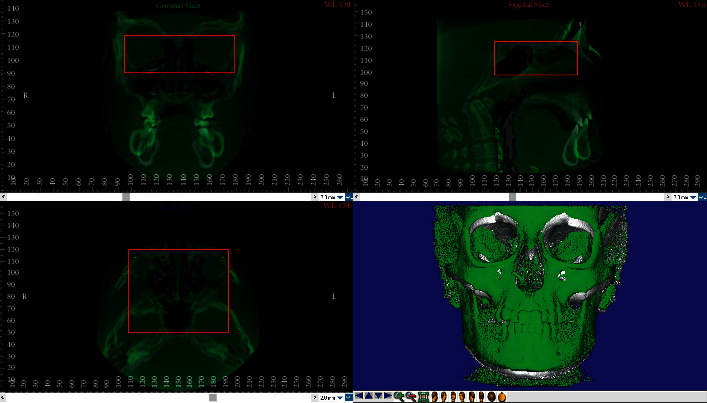
Voxel-based superimposition: selection of the Volume of Interest (VOI) on the cranial base structure of T0-CBCT (before treatment) and registration of the T1-CBCT (posttreatment).

**Figure 5:**
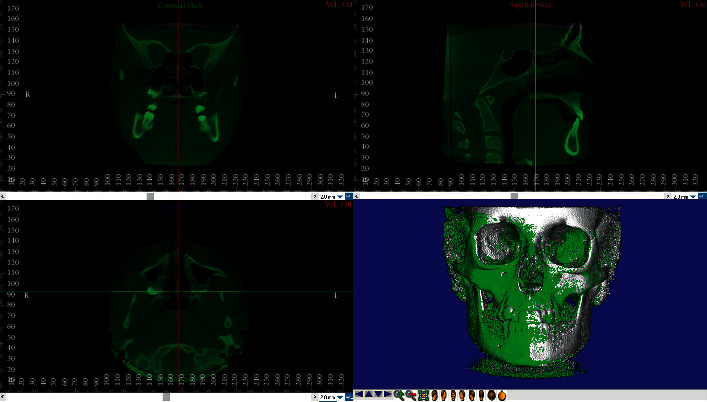
Voxel-based superimposition completed.

The voxel-based registration (VBR) is an automatic superimposition technique firstly proposed by Planché et al. [25, 38] involving the matching between the greyscale values of the voxels within the selected Volume of Interest (VOI) of two or more volumetric datasets (i.e., CBCT and CT) (Figures 4 and 5). One of the most important advantages of this technique is that, unlike landmark- and surface-based registration, it does not depend on the accuracy of landmark identification or on errors in the surface segmentation process. In fact, it is a completely automated registration method which avoids observer-dependent errors. Nowadays, there are two software applications available for performing voxel-based registration, i.e., Dolphin and Slicer 3D, both of them showing both high accuracy and reliability [17, 18, 39, 40]. This technique was firstly proposed for identifying overall treatment outcomes and different patterns of remodeling following orthognathic surgery in non-growing subjects using as reference structure the anterior cranial base [18, 38]. Afterwards, it was used for assessing the facial overall treatment outcomes (i.e., maxillary and/or mandibular displacement) in growing subjects [41] and proposed for regional registration on maxillary or mandibular bone by different authors. Ruellas et al. [17] compared different mandibular reference regions for analyzing the changes in dental arches and/or condyle/rami complex due to orthodontic treatment. Koerich et al. [22] described a voxel-based registration for maxillary and mandibular region as alternative to the anterior cranial base. Nada et al. [42] compared the accuracy and reliability of voxel-based registration using the zygomatic arch instead of the anterior cranial base in smaller FOV CBCT, concluding that the zygomatic arch can be used as stable structure. Previous studies [43, 44] compared landmark- and voxel-based registration in order to evaluate changes in mandibular condyles morphology after maxillofacial surgery [45].

Based on studies comparing the three registration methods previously cited, landmark-based superimposition is the least reliable due to the need of manual landmark selection by the operator and the lack of precise definition of 3D coordinates of cephalometric landmarks. Surface-based registration and voxel-based registration have been widely validated, the voxel-based registration being associated with less variability in terms of accuracy and reliability. In this regard, surface-based registration preliminary requires a precise segmentation of the investigated structures, which can introduce biases in the superimposition method. It was also suggested that initial approximation of the images is an important step to reduce registration time of the software and improve the precision of the superimposition.

## 3. Conclusions

Considering the continuous update in 3D imaging, clinicians must get familiar with these new technologies that have opened new scenario in the diagnosis and treatment plan of patients involved in orthodontic and maxillofacial therapies.

## Data Availability

The data used to support the findings of this study are available from the corresponding author upon request.
